# Patient reported outcome measures in Waldenström macroglobulinaemia: A real‐world data analysis from the WMUK Rory Morrison Registry

**DOI:** 10.1002/jha2.640

**Published:** 2022-12-29

**Authors:** Jahanzaib Khwaja, Encarl Uppal, Sotirios Bristogiannis, Helen McCarthy, Jaimal Kothari, Ali Rismani, Harriet Scorer, Jane Nicholson, Dima El‐Sharkawi, Shirley D'Sa, Charalampia Kyriakou

**Affiliations:** ^1^ Department of Haematology University College London Hospitals NHS Foundation Trust London UK; ^2^ Department of Haematology Royal Bournemouth Hospital Bournemouth UK; ^3^ Department of Haematology Oxford University Hospitals NHS Foundation Trust Oxford UK; ^4^ WMUK Cheshire UK; ^5^ Department of Haematology The Royal Marsden Hospital NHS Foundation Trust London UK

**Keywords:** lymphoid malignancies, quality of life, Waldenstrom macroglobulinaemia

## Abstract

Waldenström macroglobulinaemia (WM) is an incurable chronic B‐cell malignancy, but highly responsive to treatment. Treatments include fixed‐duration chemotherapy and continuous oral chemoimmunotherapy. In this expanding field, it is important to have reliable information on the impact of the various therapies on patients’ quality of life (QoL). Patient reported outcome measures (PROMs) are increasingly recognised as important to understand patient experience of disease beyond traditional clinical outcome measures. Four QoL questionnaires (EORTC QLQ‐C30 [European Organisation for Research and Treatment of Cancer quality of life core questionnaire], BIPQ [Brief Illness Perception Questionnaire], HADS [Hospital Anxiety and Depression Scale], EQ‐5D‐5L [EuroQoL 5‐dimensional descriptive system questionnaire]) are embedded in the UK national WM registry, the Rory Morrison Registry. We reviewed the results from a snapshot of PROMs. As of November 2021, 155 patients completed PROM data with 98% completion rate across all 58 questions. Complete clinical information was available for 52 patients. The majority of QoL questions (69%) failed to elicit a notable median response. Only four questions elicited statistically significant responses when comparing groups, and these were exclusively found in the EuroQoL‐5D‐5L and HADS questionnaires. Our data suggest that widely used questionnaires may not be suitable for patients with WM. We advocate the development of WM‐specific outcome measures to overcome this.

## 1 INTRODUCTION

Waldenström macroglobulinaemia (WM) is a rare chronic B‐cell lymphoproliferative disorder characterised by immunoglobulin M (IgM) monoclonal gammopathy and bone marrow infiltration by lymphoplasmacytic lymphoma [1]. WM is an incurable disease but highly responsive to treatment. Minimally symptomatic patients may be candidates for observation; however, a proportion will require treatment due to progressive lymphoma or immunological and biochemical manifestations due to the underlying monoclonal protein [2]. Treatments include fixed‐duration chemotherapy and continuous oral chemoimmunotherapy supported by clinical trial data [3, 4]. Potential future therapies include molecular targeted treatment, CAR‐T cells, immunotherapies and radiopharmaceutical approaches. In this expanding field, it is important to have reliable information on the impact of various therapies on patients’ quality of life (QoL).

Patient reported outcome measures (PROMs) are increasingly recognised as important to understand patient experience of disease beyond traditional clinical outcome measures such as progression‐free and overall survival cited in clinical trials. Less than 10% of lymphoma trials sponsored by National Cancer Institute employed PROMs [5]. PROMs may inform our view of tolerability of therapy beyond those typically reported by common terminology for adverse events and potentially delineate long‐term toxicities.

The Rory Morrison Registry is a national comprehensive data repository in the UK developed to ascertain real‐world data for patients with WM and its associated disorders [6]. Research ethics approval was obtained from the London—South East Research Ethics Committee (REC: 17/LOLO/1666). Following the launch of the clinical registry, four widely used PROM questionnaires were electronically embedded in the registry: EORTC QLQ‐C30 (European Organisation for Research and Treatment of Cancer quality of life core questionnaire) [7], BIPQ (Brief Illness Perception Questionnaire) [8], HADS (Hospital Anxiety and Depression Scale) [9] and EQ‐5D‐5L (EuroQoL 5‐dimensional descriptive system questionnaire) [10]. These questionnaires were selected due to their widespread acceptance in the setting of haematological cancers. They contained 58 questions and were distributed digitally on a quarterly basis to patients registered for PROMs. The discrepancy between the large denominator of patients on the registry with clinical data and the small number of PROM entries relates to the different routes of enrolment—the former through enrolment of clinical centres [6] and the latter via the direct recruitment of patients who specifically registered and consented for PROMs. Here, we reviewed the results from a snapshot of PROM questionnaires used to assess patient QoL in WM.

## 2 RESULTS AND DISCUSSION

As of November 2021, 1188 patients with WM were enrolled in the Rory Morrison Registry. A total of 208 patients registered to input PROM data, of which 155 (75%) completed PROM data. The data completion rate was 98% across all questions.

Of 155 patients (male 87, female 68), the median age at diagnosis was 60 years (range 33–77), and the median age at data entry was 69 years (range 42–88), with 39% (60/155) aged ≤65 years. Five percent (8/155) had a WM‐associated disorder (neuropathy, cold agglutinin disease, cryoglobulinaemia, systemic light‐chain amyloidosis or Schnitzler syndrome).

Complete clinical information in addition to PROMs was available in 52 patients. The median total therapy lines was 1 (range 0–7), half (26/52) had ≤1 total treatments and half had ≥2 therapy lines. At data entry, 35/52 (67%) were not currently on treatment. Of those actively undergoing chemoimmunotherapy, 76% (13/17) were taking an oral Bruton's tyrosine kinase inhibitor (BTKi), whilst 24% (4/17) were undertaking a chemotherapy‐based regimen.

Sixty‐nine percent of questions across all QoL questions elicited a median response that was not notable (scored 0/‘not at all’). Subgroup analysis was conducted on 31% (18/58) of questions, which identified a notable median (Table 1). The responses were analysed by age, gender, presence of WM‐associated disorder, time to diagnosis and treatment details. Of 18 questions, only four questions elicited statistically significant responses when comparing groups and these were exclusively found in the EuroQoL‐5D‐5L and HADS questionnaires (Table 2). No questions elicited significant differences in EORTC QLQ C30 or BIPQ.

**TABLE 1:** Questions and responses for 18 quality of life (QoL) questions that elicited a notable result (*n* = 155)

Question		Median (range)	Scale
EuroQoL‐5D‐5L			
1.	Your own health state today	75 (15–100)	0–100: 0 = The worst health you can imagine, 100 = The best health you can imagine
2.	Mobility	1 (1–4)	1–5: 1 = I have no problems in walking about, 5 = I am unable to walk about
3.	Usual activity	1 (1–5)	1–5: 1 = I have no problems doing my usual activities, 5 = I am unable to do my usual activities
4.	Pain/discomfort	2 (1–5)	1–5: 1 = I have no pain or discomfort, 5 = I have extreme pain or discomfort
HADS			
5.	HADS anxiety	5 (0–19)	0–21: 0–7 = Normal, 8–10 = Borderline abnormal (borderline case), 11–21 = Abnormal (case)
6.	HADS depression	4 (0–15)	0–21: 0–7 = Normal, 8–10 = Borderline abnormal (borderline case), 11–21 = Abnormal (case)
EORTC QLQ C30			
7.	How would you rate your overall quality of life during the past week?	5 (1–7)	1–7: 1 = Very poor, 7 = Excellent
8.	How would you rate your overall health during the past week?	5 (2–7)	1–7: 1 = Very poor, 7 = Excellent
9.	Have you had trouble sleeping?	2 (1–4)	1–4: 1 = Not at all, 4 = Very much
10.	Has your physical condition or medical treatment interfered with your social activities?	2 (1–4)	1–4: 1 = Not at all, 4 = Very much
11.	Were you tired?	2 (1–4)	1–4: 1 = Not at all, 4 = Very much
12.	Did you need to rest?	2 (1–4)	1–4: 1 = Not at all, 4 = Very much
13.	Have you felt weak?	2 (1–4)	1–4: 1 = Not at all, 4 = Very much
BIPQ			
14.	How much control do you feel you have over your illness?	4 (0–10)	0–10: 0 = Absolutely no control, 10 = Extreme amount of control
15.	How well do you feel you understand your illness?	8 (0–10)	0–10: 0 = Do not understand at all, 10 = Understand very clearly
16.	How concerned are you about your illness?	6 (0–10)	0–10: 0 = Not at all concerned, 10 = Extremely concerned
17.	How much do you experience symptoms from your illness?	3 (0–10)	0–10: 0 = No symptoms at all, 10 = Many severe symptoms
18.	How much does your illness affect your life?	5 (0–10)	0–10: 0 = No affect at all, 10 = Severely affects my life

**TABLE 2:** Median responses of 18 quality of life (QoL) questions

		QoL questions																	
		1	2	3	4	5	6	7	8	9	10	11	12	13	14	15	16	17	18
	*n*	EuroQoL‐5D‐5L				HADS		EORTC QLQ C30							BIPQ				
Entire group																			
Age (years)																			
≤65	60	73	1	1	2	6	5	5	5	2	2	2	2	2	4	8	6.5	3	6
>65	95	79.5	1	1	2	4	4	5	6	2	2	2	2	1	4	8	6	3	5
*p*‐Value						0.01													
Gender																			
Male	87	80	1	1	2	4	4	5	5	2	2	2	2	2	5	8	6	3	6
Female	68	73.5	1	1	2	5	5	5	5	2	2	2	2	2	4	8	6	3	5
*p*‐Value						0.01													
WM‐associated disorder																			
Yes	8	71	1	1	2	6.5	3.5	5	6	2	2	2	2	2	6	8	7.5	5	7
No	147	75	1	1	2	4.5	4	5	5	2	2	2	2	2	4	8	6	3	5
Clinical details																			
Years since diagnosis																			
≤5 years	23	73	1	2	2	6	5	5	6	2	2	2	2	2	4	8	7	3	6
>5 years	42	74	1	1	2	4	3	5	5	2	2	2	2	2	5	8	6	4	6
Present treatment status																			
On treatment	17	76	1	1	1	5.5	2	6	6	2	2	1	2	1	6	8	6	4	5
Off treatment	35	73	1	2	2	4	5	5	5	2	2	2	2	2	4	8	6	4	7
*p*‐Value				0.02			0.01												
Current treatment																			
BTKi	13	77.5	1	1	1	4.5	1	6	6	2	2	1	2	1	6	8	6	3	3
Chemotherapy	4	53.5	1	2	1	6	5	5.5	5	2.5	2	1.5	1	2	2	9	9	6	6.5
*p*‐Value							0.03												

Abbreviations: BTKi, Bruton's tyrosine kinase inhibitor; WM, Waldenström macroglobulinaemia.

On global assessment, the median across all scores for HADS was in the ‘normal’ range (0–7 score) for levels of anxiety or depression. Symptom scores across EuroQoL‐5D‐5L and functional scales in EORTC QLQ C30 were normal or minimally affected in the majority of all questions. Forty percent of patients felt that they had minimal control (score 0–3) over their illness on BIPQ. Overall, 24% and 22% experienced borderline or abnormal anxiety or depressive symptoms on HADS, respectively. HADS anxiety score was higher amongst younger patients (aged ≤65 years vs. >65 years, *p* = 0.01) and females (*p* = 0.01). The HADS depression scores were higher in those not currently on treatment (*p* = 0.01), and for those on treatment, scores were higher in those on chemotherapy compared with oral BTKi (*p* = 0.03). Usual activity was perceived to be more affected in those off active treatment (*p* = 0.02) and mobility perceived to be impacted more greatly in those with fewer total lines of therapy (≤1 vs. ≥2, *p* = 0.02) in the EuroQoL‐5D‐5L, although numbers were small in these subgroups. There was no observable difference in other groups, including the small numbers of patients with WM‐associated condition.

Our cohort demonstrates that the majority of the QoL questions (69%) failed to elicit a notable median response. The poor ability of most questions to demonstrate meaningful values likely reflects the generalised nature of these tools. A large body of QoL data exists for Hodgkin lymphoma and is now emergent in non‐Hodgkin lymphoma [11]; however, data are scant for WM. Although a rare disorder, with improved survival and prognosis, patients with WM experience long‐term physical and psychosocial effects from disease and therapy. Our preliminary snapshot data demonstrate the feasibility of digital PROM self‐entry within national registries, with near complete completion rates. The four questionnaires employed are standardly used generic questionnaires and have been used in clinical trials in WM [4]. However, patient feedback highlighted the onerous nature of these questionnaires, emphasising the need for dedicated patient reported outcome tools that yield meaningful data. The failure of these general questions to detect meaningful issues suggests the need for the development of WM disease‐specific PROMs, as has been recently developed in other haematological malignancies, including chronic lymphocytic leukaemia [12] and chronic myeloid leukaemia [13].

Two reports of PROMs in WM have been published in the literature. The global database across 19 countries, WhiMSICAL, employed two PROM tools: EORTC QLQ‐C30, as in our cohort, and Impact of Event Scale‐6, a measure of current post‐traumatic stress symptoms. The investigators found that 10% of participants had scores consistent with post‐traumatic stress disorder (94% positive predictive value) at a median of 43 months (interquartile range 15–98) post‐diagnosis [14]. EORTC QLQ‐C30 snapshot analysis showed that patients taking BTKi had high QoL scores, with a mean global scale of 80.1 ± 16.2 (*n* = 44) versus 68.3 ± 22.6 (*n* = 57, *p* = 0.004). There was a wide variation in chemoimmunotherapies available (46 differing frontline regimens amongst 302 participants); therefore, it is difficult to compare this cohort with our data. An Italian single institute reported on PROMs in 143 patients with WM, monoclonal gammopathy of undetermined significance or an IgM‐associated disorder [15] using EORTC QLQ‐C30, HADS and EQ‐5D‐5L, as in our cohort, as well as FACT‐GOG neurotoxicity (Functional Assessment of Cancer Therapy‐Gynaecologic Oncology Group). Comparing treated, untreated and IgM‐associated disorders, no differences were found amongst most questionnaires (EORTC QLQ‐C30, HADS anxiety and depression scores or FACT‐GOG neurotoxicity score), although differences were seen in the EuroQoL‐5D visual analogue scale score with significantly worse scores in treated patients compared with untreated and IgM‐associated disorders. IgM‐related neuropathy (*n* = 19) had lower scores on QoL. IgM‐related neuropathy warrants further evaluation in PROMs.

We acknowledge the limitations in our provisional report. Completion of PROMs is limited by survivor bias, and selection bias based on participants most likely to engage with these questions is inherent. Potential confounding variables such as socioeconomic status and ethnicity were not collected and are important to elucidate. We also welcome longitudinal data to assess cumulative morbidity.

Overall, our data suggest that widely used questionnaires may not be suitable for patients with WM and may not capture the experience of patients with this disorder. We advocate the development of WM‐specific outcome measures to overcome this. WM‐tailored tools developed by patients could reflect the diverse immunological paraproteinemia complications [2]. Such tools could collect data with greater applicability and utility to clinical and research practice, potentially improving quality of care and treatment decision making.

## FUNDING INFOMATION

The authors received no specific funding for this work.

## CONFLICTS OF INTEREST

Shirley D'Sa reports speaker fees and research funding from Janssen, BeiGene and Sanofi. Remaining authors declare they have no conflicts of interest.

## ETHICS STATEMENT

Research ethics approval was obtained from the London—South East Research Ethics Committee (REC: 17/LOLO/1666).

## Data Availability

The data that support the findings of this study are available on request from the corresponding author. The data are not publicly available due to privacy or ethical restrictions.
